# Characterizing of dropouts in the mental health of refugees and asylum seekers (MEHIRA) study examining the effects of a stepped and collaborative care model – a multicentered rater-blinded randomized controlled trial – CORRIGENDUM

**DOI:** 10.1017/S0033291725000170

**Published:** 2025-03-24

**Authors:** Solveig Kemna, Max Bringmann, Carine Karnouk, Andreas Hoell, Mira Tschorn, Inge Kamp-Becker, Frank Padberg, Aline Übleis, Alkomiet Hasan, Peter Falkai, Hans-Joachim Salize, Andreas Meyer-Lindenberg, Tobias Banaschewski, Frank Schneider, Ute Habel, Paul Plener, Eric Hahn, Maren Wiechers, Michael Strupf, Andrea Jobst, Sabina Millenet, Edgar Hoehne, Thorsten Sukale, Martin Schuster, Raphael Dinauer, Nassim Mehran, Franziska Kaiser, Klaus Lieb, Andreas Heinz, Michael Rapp, Malek Bajbouj, Kerem Böge

This notice is to highlight that one of the affiliations in this article was incorrectly published as: ‘Department of Psychiatry and Psychotherapy, Psychosomatics and Psychotherapy, Faculty of Human Medicine, Philipps-University Marburg, Marburg, Germany’.

This affiliation should have been written as: ‘Department of Child and Adolescent Psychiatry, Psychosomatics and Psychotherapy, Faculty of Human Medicine, Philipps-University Marburg, Marburg, Germany’.

This has now been updated in the HTML and PDF versions of the manuscript.

The authors apologize for this error.

## References

[r1] Kemna S, Bringmann M, Karnouk C, et al. (2024). Characterizing of dropouts in the mental health of refugees and asylum seekers (MEHIRA) study examining the effects of a stepped and collaborative care model – a multicentered rater-blinded randomized controlled trial. Psychological Medicine, 54(16), 4868–4877. 10.1017/S0033291724003179 39773258

